# Evaluating Power and Type 1 Error in Large Pedigree Analyses of Binary Traits

**DOI:** 10.1371/journal.pone.0062615

**Published:** 2013-05-03

**Authors:** Anna C. Cummings, Eric Torstenson, Mary F. Davis, Laura N. D’Aoust, William K. Scott, Margaret A. Pericak-Vance, William S. Bush, Jonathan L. Haines

**Affiliations:** 1 Center for Human Genetics Research, Vanderbilt University, Nashville, Tennessee, United States of America; 2 Dr. John T. Macdonald Foundation Department of Human Genetics and John P. Hussman Institute of Human Genomics, Miller School of Medicine, University of Miami, Miami, Florida, United States of America; Centers for Disease Control and Prevention, United States of America

## Abstract

Studying population isolates with large, complex pedigrees has many advantages for discovering genetic susceptibility loci; however, statistical analyses can be computationally challenging. Allelic association tests need to be corrected for relatedness among study participants, and linkage analyses require subdividing and simplifying the pedigree structures. We have extended GenomeSIMLA to simulate SNP data in complex pedigree structures based on an Amish pedigree to generate the same structure and distribution of sampled individuals. We evaluated type 1 error rates when no disease SNP was simulated and power when disease SNPs with recessive, additive, and dominant modes of inheritance and odds ratios of 1.1, 1.5, 2.0, and 5.0 were simulated. We generated subpedigrees with a maximum bit-size of 24 using PedCut and performed two-point and multipoint linkage using Merlin. We also ran MQLS on the subpedigrees and unified pedigree. We saw no inflation of type 1 error when running MQLS on either the whole pedigrees or the sub-pedigrees, and we saw low type 1 error for two-point and multipoint linkage. Power was reduced when running MQLS on the subpedigrees versus the whole pedigree, and power was low for two-point and multipoint linkage analyses of the subpedigrees. These data suggest that MQLS has appropriate type 1 error rates in our Amish pedigree structure, and while type 1 error does not seem to be affected when dividing the pedigree prior to linkage analysis, power to detect linkage is diminished when the pedigree is divided.

## Introduction

Complex pedigrees from isolated populations have gained popularity for genetic studies due to their pedigree size, genetic homogeneity, and environmental homogeneity [1]–[3]. Despite these advantages, pedigree size and genetic homogeneity complicate analyses and can make results difficult to interpret. Association analyses must correct for the genetic relatedness of individuals (kinship) within families. Simple family-based association analyses for binary traits, such as TDT [4], PDT [5], [6], and FBAT [7] do not accommodate large consanguineous pedigrees. To accommodate large and complex pedigree structures, other methods have been developed including the CC-QLS (case control quasi-likelihood score) [8] and MQLS (modified quasi-likelihood score) [9] that use the kinship coefficients of all pairs of pedigree members to accurately define and correct for all relationships when testing for association. These tests also allow genetic linkage to contribute to the statistics. The MQLS is an extension and improvement of the CC-QLS and is able to use phenotype data of samples without genotypes and can also differentiate between and incorporate unaffected controls and controls of unknown phenotype in the analysis. Thornton and McPeek evaluated type 1 error and power in relatively simple simulated pedigrees, showing that type 1 error was not inflated and that power was relatively good [9]. The most complex pedigree they sampled contained 154 individuals spanning five generations. Our Amish pedigree contains 4,998 members (798 genotyped) spanning 13 generations [3]. To our knowledge, simulations from a pedigree structure typical of a large founder population, such as the Amish, have not been performed. Therefore, we still have limited understanding of how these tests perform on a larger founder pedigree.

Pedigree size and complexity also present problems when running linkage analyses because even the best available linkage programs, such as Allegro [10], [11], Vitesse [12], Superlink [13]–[15], and Merlin [16], can only handle pedigrees under a certain size and complexity threshold. Programs based on the Elston-Stewart algorithm (Vitesse [12], Superlink [13]–[15]) are limited in both the pedigree complexity and number of markers, whilst programs based on the Lander-Green algorithm (Allegro [10], [11] and Merlin [16]) can handle a whole chromosome of markers but require simpler pedigrees. Therefore, it is necessary to split the pedigree into smaller sub-pedigrees when performing linkage analysis on a data set exceeding these limitations. One method for doing this is PedCut [17], which generates sub-pedigrees with the maximal number of subjects of interest within a specified bit-size (two times the number of non-founders minus the number of founders [18]) limit conducive to two-point and multipoint linkage analyses. Dividing the pedigree alters the overall flow of alleles detected by the linkage analysis, and could alter results [19], [20].

GenomeSIMLA [21] is a forward-time population-based simulation package for generating large-scale SNP data in both case-control and family-based designs and has been adapted to efficiently produce SNP data in any pedigree structure given a pedigree template. We used this extended version of GenomeSIMLA to evaluate the power and false-positive rates for association and linkage analyses for an Amish pedigree structure. Using data simulations, we evaluated the false-positive rates generated by two-point and multipoint linkage using Merlin following the use of PedCut. We also assessed the effect of splitting pedigrees on type 1 error rates is to run MQLS on the sub-pedigrees to compare the results to an MQLS analysis of the unified pedigree. By generating simulated data based on the true pedigree structure of our cohort, we can more accurately estimate power and false positive rates specific to our data set, and the same techniques could be applied for other complex pedigrees.

## Methods

### Simulations

We extended the software package GenomeSIMLA to generate complex pedigree structures based on a template pedigree. Once a population of chromosomes has been created, a collection of founders is drawn and are mated based on the pedigree structure to produce all generations of the pedigree. Affection status is assigned by applying a penetrance function with the option of only assigning known phenotype and genotype data to the same individuals with known phenotype and genotype data in the template pedigree, maintaining a more realistic distribution of genotyped affected and unaffected individuals in the pedigree. We simulated a null disease model into 1000 pedigree replicates, each with 124 autosomal SNPs with a spacing of 0.062 centimorgans and no linkage disequilibrium between them, using our recently published 4998-member Amish pedigree with almost identical affection status (798 genotyped, 106 affected) [3]. The Anabaptist Genealogy Database provided the pedigree structure [22]. The minor allele frequency (MAF) was set to 0.2, to approximate the mean MAF in the recent GWAS study of our Amish pedigree [3].

For studies of power, we modified the null simulation, forcing one of the 124 SNPs to have either a dominant, recessive, or additive effect with odds ratios of 1.1, 1.5, 2.0, or 5.0 for this locus, resulting in 12 total disease models. The minor allele frequency for the ‘disease’ SNP was held constant at 0.2, consistent with the GWAS hypothesis of at least one common variant increasing risk of a common disease. One thousand replicates were simulated for each disease model.

### Analyses

We ran MQLS (software version 1.2) to test for association and used option ‘1’ to include all individuals, cases, controls, and individuals with unknown phenotype in the analyses. More recent versions (starting at version 1.5) of MQLS include a more robust variance estimator [23], which was not implemented in these analyses but would not likely make a significant difference in our results. We tallied the number of p-values below values of interest in each of the replicates. For the type 1 error study any p-value below the threshold was included in the count, and for the power studies any p-value below the threshold at the ‘disease’ SNP was counted. The average number of p-values was then calculated across each set of 1000 replicates.

To generate sub-pedigrees within a bit-size limit of 24, we ran PedCut [17] in each of the simulated pedigrees using affected individuals and unaffected siblings of the affected individuals as subjects of interest. We ran two-point and multipoint parametric and nonparametric linkage analyses on the PedCut pedigrees using Merlin [16]. Multipoint linkage was run on all 124 markers. Parametric HLOD scores were computed assuming affecteds-only autosomal dominant and recessive models of 0 penetrance for no disease allele and 0.0001 for 1 or 2 copies of the disease allele under the dominant model, and penetrances of 0 for 0 or 1 disease allele and 0.0001 for 2 disease alleles under the recessive model. A disease allele frequency of 1% was used to mimic our recently published genome-wide study and to approximate the expected disease allele frequency in the general Amish population. We note a typographical error in that paper which misreported the disease allele frequency to be 10% [3]. Semi-parametric LOD scores (LOD*) were computed using the NP-all and NP-pairs statistics. For the two-point type 1 error results, we tallied the number of SNPs out of the 124 simulated SNPs with HLOD/LOD scores above certain thresholds. We averaged these tallies across the 1000 replicates and divided by 124. For two-point power analyses we tallied the number of times the disease SNP crossed the HLOD/LOD threshold in each set of 1000 replicates. For type 1 error and power evaluations of multipoint linkage analysis, we tabulated the maximum parametric HLOD and nonparametric LOD of each replicate and calculated the percentage of the peak HLOD/LOD scores that crossed thresholds. We allowed the maximum HLOD/LOD to be at any of the 124 SNPs since we simulated each replicate to be similar to a region in our previous multipoint study (3) and we do not expect the peak to always be precisely at the disease SNP every time.

We also ran MQLS on the sub-pedigrees to compare those results to running MQLS on the unmanipulated large simulated pedigrees. Prior to running MQLS, we re-calculated kinship coefficients using the sub-pedigree structures rather than the entire pedigree structure to model some of the effect of losing the entire pedigree structure that might occur when using association to follow-up linkage analysis in these sub-pedigrees. We determined type 1 error rates and power as before.

All computations were performed using either the Center for Human Genetics Research (CHGR) computational cluster or the Advanced Computing Center for Research and Education (ACCRE) cluster at Vanderbilt University. Scripts and pedigree structures are available upon request.

## Results

### MQLS

In 1000 runs of MQLS, each with the entire 4998-member pedigree and 124 null SNPs, we see average type 1 error rates of 5.06%, 1.02%, 0.56%, and 0.13% associated with p-values less than 0.05, 0.01, 0.005, and 0.001, respectively. Therefore, we do not see an inflated type 1 error rate when running MQLS in our pedigree structure.

Evaluating power, we find, as expected, that we have the least power to detect association when the underlying disease model is recessive and the most power to detect association when the underlying disease model is additive. For dominant and additive models we have >90% power to detect association at p≤0.05 when the simulated odds ratio is at least 2.0, but power drops significantly at an odds ratio of 1.5. With a very strong effect of OR = 5, we have very high power to detect association even as low as a p-value of 5.0E-8 (such as would be needed for Bonferroni-corrected GWAS). Under the recessive models power was only >80% using a p-value threshold of 0.05 for an odds ratio of 5.0 (Table 1).

**Table 1:** Average percentage of times (power) per model disease SNPs was under p-value thresholds when running MQLS on whole simulated pedigrees.

Disease Model, Odds Ratio	%≤0.05	%≤5E-3	%≤5E-4	%≤5E-5	%≤5E-6	%≤5E-7	%≤5E-8
recessive, OR 1.1	6	0	0	0	0	0	0
recessive, OR 1.5	12	4	1	0	0	0	0
recessive, OR 2.0	26	9	3	1	0	0	0
recessive, OR 5.0	**87**	75	61	48	38	29	21
dominant, OR 1.1	8	2	0	0	0	0	0
dominant, OR 1.5	50	23	9	3	1	1	0
dominant, OR 2.0	**92**	72	47	28	13	7	4
dominant, OR 5.0	**100**	**100**	**100**	**100**	**100**	**99**	**98**
additive, OR 1.1	11	3	0	0	0	0	0
additive, OR 1.5	67	36	19	8	3	1	1
additive, OR 2.0	**96**	**87**	69	50	33	20	12
additive, OR 5.0	**100**	**100**	**100**	**100**	**100**	**100**	**100**

Power ≥80% in bold.

### MQLS-PedCut

Using the same sets of pedigrees, but dividing them into subpedigrees using PedCut, the type 1 error rates when running MQLS hardly changed from the MQLS analysis using whole pedigrees. The type 1 error rates were 5.16%, 1.06%, 0.51%, and 0.11% for the same p-value thresholds.

On the other hand, evaluating power when subdividing the pedigree before running MQLS we do see a loss of power. Power is only >80% for dominant and additive models at an odds ratio of 5.0 (Table 2).

**Table 2:** Average percentage of times (power) per model disease SNPs was under p-value thresholds when running MQLS on subdivided simulated pedigrees.

Disease Model, Odds Ratio	%≤.05	%≤5E-3	%≤5E-4	%≤5E-5	%≤5E-6	%≤5E-7	%≤5E-8
recessive, OR 1.1	6	0.5	0	0	0	0	0
recessive, OR 1.5	8	1	0.4	0.1	0	0	0
recessive, OR 2.0	15	3	0.6	0.1	0	0	0
recessive, OR 5.0	74	51	34	19	10	5	2
dominant, OR 1.1	8	0.3	0	0	0	0	0
dominant, OR 1.5	24	5	1	0.2	0	0	0
dominant, OR 2.0	55	21	7	2	0.6	0.1	0
dominant, OR 5.0	**99**	**90**	72	49	27	13	6
additive, OR 1.1	6	0.6	0	0	0	0	0
additive, OR 1.5	33	9	2	0.1	0	0	0
additive, OR 2.0	70	37	16	5	2	0.8	0
additive, OR 5.0	**100**	**98**	**92**	**80**	65	43	24

All numbers are percentages. Power ≥80% in bold.

### Two-point Linkage

Averaging across 1000 replicates of two-point parametric linkage analysis using sub-pedigrees with a bit-size ≤24, we see low type 1 error rates, which were nearly the same when running dominant and recessive models. The type 1 error rate using a critical value of HLOD of 3 under the dominant model was only 0.01% and under the recessive model was only 0.02%. Nonparametric analyses had no type 1 error at LOD threshold of 2 and 3 (table 3).

**Table 3:** Percentage of SNPs (type 1 error) above HLOD thresholds using PedCut followed by two-point parametric linkage analyses assuming dominant and recessive models and nonparametric linkage analysis using the ‘all’ and ‘pairs’ statistics.

	HLOD/LOD >1	HLOD/LOD >2	HLOD/LOD >3
dominant	2.21%	0.18%	0.01%
recessive	2.02%	0.20%	0.02%
NPL all	0.15%	0	0
NPL pairs	0.05%	0	0

According to our simulations, we had >80% power to detect a two-point HLOD ≥1.0 with a simulated additive model with OR = 5.0 when a dominant model is assumed during linkage analysis. All other circumstances had <80% power; however, with the simulated dominant model with OR = 5, Merlin was able to detect the disease SNP almost 80% of the time at or above an HLOD of 1 when a dominant model was assumed. Even when a recessive model was assumed two-point linkage analysis was not powerful for any of the simulated recessive scenarios. Parametric analyses were more powerful than nonparametric analyses (table 4).

**Table 4:** Percentage of times (power) disease SNP crossed parametric HLOD or nonparametric LOD thresholds using PedCut followed by Merlin two-point parametric and nonparametric linkage analyses.

	HLOD/LOD ≥1.0				HLOD/LOD ≥2.0				HLOD/LOD ≥3.0			
Model, Odds Ratio	Dom	Rec	All	Pairs	Dom	Rec	All	Pairs	Dom	Rec	All	Pairs
dominant, OR 1.1	2.4	2.3	0	0	0.1	0	0	0	0	0	0	0
dominant, OR 1.5	3.6	3.6	0.7	0.3	0.6	0.7	0	0	0	0	0	0
dominant, OR 2.0	8.3	9.1	2.3	0.7	1.7	1.2	0	0	0.5	0.4	0	0
dominant, OR 5.0	77.7	71	50	33.6	51.1	43.3	4.7	0.7	28.2	22.8	0	0
recessive, OR 1.1	2.6	2.6	0.4	0.1	0.4	0.4	0	0	0	0.1	0	0
recessive, OR 1.5	2.9	2.3	0.3	0.1	0.2	0.1	0	0	0	0	0	0
recessive, OR 2.0	2.4	2.3	0.2	0.1	0.2	0.2	0	0	0	0	0	0
recessive, OR 5.0	13.3	13.9	9	6.5	3.7	4.1	0.4	0.1	0.5	1.4	0	0
additive, OR 1.1	2.5	2.2	0.3	0.1	0.1	0.2	0	0	0	0	0	0
additive, OR 1.5	4.3	3.7	1	0.6	0.4	0.8	0	0	0.2	0.1	0	0
additive, OR 2.0	12.3	10.4	3	1.5	2.6	2.3	0.1	0	0.6	0.3	0	0
additive, OR 5.0	**85.5**	79.1	64.9	48.9	67.8	53.6	12.2	3.4	44	32	0.7	0

1000 replicates of each disease model were performed. All numbers are percentages. Power >80% in bold.

### Multipoint Linkage

When running multipoint analysis on the same sets of sub-pedigrees we see both higher type 1 error and higher power for most circumstances except for a simulated dominant model with OR = 5. For multipoint analyses we see higher type 1 error and power for nonparametric analyses than for parametric analyses (tables 5 and 6). For both two-point and multipoint linkage, the highest power for detecting linkage was seen with a simulated additive model with OR = 5.0 (tables 4 and 6).

**Table 5:** Percentage of SNPs (type 1 error) above parametric HLOD and nonparametric LOD thresholds using PedCut followed by multipoint parametric linkage analyses assuming dominant and recessive models and nonparametric linkage analysis using the ‘all’ and ‘pairs’ statistics.

	HLOD/LOD ≥1	HLOD/LOD ≥2	HLOD/LOD ≥3
dominant	23.9%	7.5%	2.5%
recessive	19.7%	6.8%	2.5%
NPL all	44.2%	16.5%	4.6%
NPL pairs	44.7%	16%	3.7%

**Table 6:** Power to detect parametric HLOD and nonparametric LOD thresholds using PedCut followed by multipoint parametric linkage analyses assuming dominant and recessive models and nonparametric linkage analysis using the ‘all’ and ‘pairs’ statistics.

	HLOD/LOD ≥1.0				HLOD/LOD ≥2.0				HLOD/LOD ≥3.0			
Model, Odds Ratio	Dom	Rec	All	Pairs	Dom	Rec	All	Pairs	Dom	Rec	All	Pairs
dominant, OR 1.1	22.4	18	44.1	43.2	6.9	5.4	13.7	14	2.1	1.8	3.6	2.7
dominant, OR 1.5	23.3	21.7	44.9	44.1	7.8	6.8	15.2	15	2.4	1.6	3.5	2.6
dominant, OR 2.0	26.7	22.1	48.1	47.7	8.8	6.6	17.7	16.6	1.9	1	5.7	4.7
dominant, OR 5.0	43.8	33	72.9	72.5	20.8	13.5	41.6	41.3	7.8	5.2	19.5	16.6
recessive, OR 1.1	22.8	19.7	41.6	41.6	7.6	5.3	16	15	2.2	1.7	4	2.8
recessive, OR 1.5	24.2	20.7	43.9	44.2	6.5	5.8	16.8	16.6	1.4	1.4	4.8	4.1
recessive, OR 2.0	23.2	19.7	43.9	44.6	7.5	6.1	15.1	14.7	1.9	1.8	3.5	3.2
recessive, OR 5.0	31	26.2	54.3	56.5	10.3	8.2	23.6	23.1	3.4	3.2	7.7	6.3
additive, OR 1.1	23.5	19.2	44.1	44.2	6.9	5.7	15.4	14.7	2.9	2.6	4.4	3.6
additive, OR 1.5	26	21.5	45.5	46.2	8.6	5.8	18	17.1	1.9	1.4	5.4	4.2
additive, OR 2.0	30.7	26.5	51.4	52.7	10.6	7.3	20.8	20.2	2.5	1.5	6.4	5.7
additive, OR 5.0	50.5	39.6	77.9	77.5	26.9	18.8	52	49.9	12	8	25.9	21.7

All numbers are percentages.

## Discussion

Pedigrees from population isolates provide rich datasets for genetic analyses; however, the size and complexity of the pedigrees contribute to ambiguity when running analyses and interpreting results. We have used this approach to discover novel susceptibility loci for complex diseases, such as Alzheimer disease and Parkinson’s disease, by studying the Amish communities of Ohio and Indiana [1], [3], [24]–[26]. In a recent genome-wide study using this population [3], 798 successfully genotyped individuals connected into one 13-generation, 4998-member pedigree with consanguineous loops. Using this same pedigree structure, we simulated 1000 pedigree replicates.

Simulations of pedigrees as large and as complex as an Amish pedigree and other population isolates to assess the type 1 error rate and power of MQLS have not been previously published, so we sought to fill this void. We did not see an inflated type 1 error rate in our simulated pedigrees. Therefore, MQLS is an appropriate method for analyzing pedigrees as large and as complex as the Amish. MQLS has sufficient power to detect a strong effect of OR = 5 when the mode of inheritance is recessive, dominant, and additive and a more moderate effect of OR = 2 when the mode of inheritance model is dominant or additive. While these are large effect sizes compared to those typical of complex diseases, in a homogeneous founder population a larger effect size is more likely.

Linkage analyses for a pedigree of this size and complexity require pedigree splitting, but the effect of using PedCut to subdivide the pedigrees on the type 1 error and power of linkage analysis is not known. Using a bit-size limit of 24 for sub-pedigree size (to allow analysis in Merlin), we saw a low type 1 error rate associated with an HLOD of 3.0 for both two-point and multipoint linkage (lower for two-point). An HLOD of ∼3 has traditionally been a ‘significant’ HLOD score, and the low type 1 error rate in this instance all allows us to confidently use this threshold when evaluating linkage results from the Amish sub-pedigrees. These approaches, however, were not powerful when we analyzed simulated 1-locus disease models in pedigrees with this number of variously related individuals.

Unfortunately, we cannot analyze the entire 4,998 member pedigree for linkage to compare the type 1 error and power to analyses of sub-pedigrees for linkage. We can, however, compare the type 1 error of association analysis using MQLS on the entire pedigree versus using MQLS on the sub-pedigrees. Splitting the simulated pedigrees did not affect the type 1 error when running MQLS. This result does not guarantee that splitting a pedigree will not lead to any spurious positive results, since other studies suggest otherwise (14). We do see a loss of power due to splitting the pedigrees because many pedigree connections are disrupted. In a previous simulation study McArdle et al saw that type 1 error increased but power was not affected when ignoring family structure while performing association analysis. Their conclusion was based on testing relatively simple pedigrees compared to singletons, which was a common approach at that time [27]. Our results compare results from the entire, highly complex pedigree to those from smaller but still large pedigrees.

Through these simulations we see that MQLS has acceptable type 1 error rates even when using an extremely complex pedigree structure. Type 1 error rates are also acceptable when splitting pedigrees prior to linkage analysis, consistent with a related study (13). Unfortunately, but not surprisingly, significant power is lost when pedigrees are divided. Development of new methods or extensions of current methods to use more pedigree information to perform multipoint linkage analyses or implementation of alternative methods such as identifying identical by descent (IBD) shared segments [26] would greatly improve our ability to query the rich genetic information of founder populations.
